# Airway Management in Patients With Vocal Cord Paralysis: A Review of Intubation Techniques, Intraoperative Challenges, and Outcomes

**DOI:** 10.7759/cureus.93264

**Published:** 2025-09-26

**Authors:** Rayyan Bhutta, Ali Osman, Tala Maya, Justin Ma, Ali Al Saeed, Sumit Sidhu, Mariya Wood, Bilal Akhtar, Sirimas Lau, Matthias Franzen

**Affiliations:** 1 Anesthesiology, Ohio University Heritage College of Osteopathic Medicine, Dublin, USA; 2 Anesthesiology, University of Louisville School of Medicine, Louisville, USA; 3 Anesthesiology, California Health Sciences University College of Osteopathic Medicine, Clovis, USA; 4 Anesthesiology, Lake Erie College of Osteopathic Medicine, Greensburg, USA; 5 Anesthesiology, California State University, Fresno, Fresno, USA; 6 Anesthesiology, The Ohio State University, Columbus, USA; 7 Anesthesiology, Otterbein University, Westerville, USA; 8 Anesthesiology, OhioHealth Doctors Hospital, Columbus, USA

**Keywords:** airway techniques, difficult airway intubation, difficult airway management, management of vocal cord paralysis, perioperative care

## Abstract

Vocal cord paralysis (VCP) presents with unique challenges when managing airways, particularly during endotracheal intubation. This literature review explores the effects of unilateral and bilateral VCP on intubation technique, first-pass success, extubation safety, and perioperative airway planning. While video laryngoscopy (VL) is becoming increasingly favored for its superior airway visualization, it does not always confirm successful placement in patients with VCP. Complications such as airway edema, aspiration risk, and cuff misplacements are increased in this patient population, and they may be more vulnerable to post-extubation complications, especially without the use of steroid prophylaxis or intensive post-operative planning. A comparison of direct laryngoscopy (DL) versus VL in this patient population reveals a gap in the literature, emphasizing the need for more research in airway management in patients with VCP. This review combines current practices, clinical considerations for anesthesia providers, and identifies areas for further investigation.

## Introduction and background

In anesthetic airway management, vocal cord paralysis (VCP) introduces a unique set of difficulties, many of which are still not adequately recognized in both current research and standard practice [1,2]. Often caused by iatrogenic injury during thyroid, cervical spine, or cardiothoracic surgeries, VCP can be unilateral or bilateral and can also develop from idiopathic causes, neurological diseases, or cancer [3,4]. The three pillars of safe anesthetic care - phonation, breathing, and airway protection - are all disrupted by reduced vocal fold motion, regardless of its cause. Particularly during sedation or after extubation, the paralysis increases the risk of aspiration and airway obstruction by decreasing protective laryngeal reflexes, changing airflow dynamics, and decreasing the efficiency of coughing [5,6].

Until glottic aperture restrictions and decreased compensation are revealed by airway manipulation, unilateral paralysis may remain undetected. Especially during induction or emergence when neuromuscular tone fluctuates, bilateral VCP is more serious and commonly results in stridor, dyspnea, and near-complete airway obstruction [7]. Acute airway failure can occur in these patients even in cases of minor edema or inflammation. However, preoperative voice evaluation and laryngeal imaging are not frequently included in airway evaluations, particularly when performing non-head-and-neck surgeries, in spite of these established risks [8].

Due to enhanced laryngeal visibility, video laryngoscopy (VL) is often believed to alleviate airway issues; however, in individuals with VCP, its benefits may be deceptive. VL makes it easier to see the glottis, but it does not guarantee that the intubation will be successful, particularly if the vocal cords are stuck in an irregular or constricted posture [9]. VCP increases the chance of paralyzed cord damage, cuff misalignment, esophageal misplacement, and false assurance in a patent airway, according to studies [10]. In addition, subglottic constriction, which might still result in resistance during tube passage or jeopardize ventilation following intubation, is not visible via glottic imaging.

Patients with VCP are at especially high risk during extubation. Laryngospasm, post-extubation stridor, and silent aspiration all become probable through reduced glottic closure, impaired laryngeal feeling, and weakened reflexes [2]. After being extubated, bilateral VCP patients may rapidly decompensate and frequently need tracheostomy, reintubation, or prolonged intensive care unit monitoring. Nevertheless, there are no VCP-specific extubation protocols in place. Patients are at risk for avoidable respiratory episodes due to the uneven application of post-extubation laryngeal checks, cuff leak testing, and prophylactic steroids [10].

The absence of VCP-specific guidelines in national difficult airway algorithms, including those provided by the American Society of Anesthesiologists, adds to the problem. Few studies have thoroughly assessed intubation procedures, extubation tactics, or postoperative outcomes in the VCP population, which indicates a shortage of studies as well as a lack of awareness [2,7]. Rarely are even high-risk surgical patients assessed for VCP before surgery, which can result in missed diagnosis and unexpectedly challenging airways [1].

Underestimating VCP has severe implications. The clinical, emotional, and financial effects are substantial, and this group is significantly more likely to experience compromised airways, extended intubation, vocal fold damage, and postoperative aspiration [3,8]. In addition, a partial VCP might develop into a bilateral one due to surgical damage to the recurrent laryngeal nerve during intubation or operational manipulation, transforming a challenging but controllable airway into a potentially fatal circumstance [4].

In this review, we review the most current research on intubation success, extubation safety, and perioperative decision-making, in addition to the pathophysiological consequences of VCP on airway management. Furthermore, we draw attention to the fact that VCP is not included in a large number of airway algorithms and emphasize the critical necessity for focused study in addition to clinical awareness. A high-risk airway is only manageable when it is anticipated; in the case of VCP, however, it is often not.

## Review

Methods

A focused literature review was conducted from February 2025 to May 15, 2025. PubMed/MEDLINE, Embase, Scopus, Web of Science, the Cochrane Library, and Google Scholar were searched using the terms “vocal cord paralysis,” “vocal fold paralysis,” “recurrent laryngeal nerve injury,” “laryngeal immobility,” “airway,” “intubation,” “video laryngoscopy,” “fiberoptic intubation,” “endotracheal tube,” “bougie,” “cuff leak,” “extubation,” “stridor,” “aspiration,” “steroids,” and “tracheostomy,” limited to human adults, the English language, and publication dates from 1985 through April 2025. Reference lists of included articles and relevant society guidance, including difficult airway guidelines, were hand searched to identify additional studies. All authors independently screened and reviewed articles, extracted data, and developed core themes; discrepancies were resolved by consensus, and the team synthesized a narrative review aligned with the study objectives.

Pathophysiology and airway implications

VCP can significantly alter the anatomy and physiology of the larynx on both neuromuscular and structural levels. This can have important implications in terms of both airway patency and airway protection. Synkinesis or abnormal reinnervation of the recurrent laryngeal nerve can result in bilateral activation of the adductors and abductors, resulting in paradoxical vocal fold movement or incompletely open vocal folds [11,12]. While the unilateral effects of VCP can sometimes be overcome, any distortion of anatomy can make intubation difficult if VCP is not recognized before induction [1,13]. Bilateral VCP or limited abduction of one or both vocal folds, on the other hand, results in both cords being fixed close to the midline and hence can greatly decrease the glottic aperture. This can make the airway vulnerable to obstruction, especially during induction or emergence, as normal neuromuscular tone is lost [7,14]. Similarly, a lack of glottic motion can impair airway clearance and predispose to aspiration, airway edema, and mucosal injury [15,16]. These effects may be more prominent in pediatric and toxicologic patients, where early airway compromise and recurrent lower respiratory tract infections are more prevalent. Posterior prolapse of the arytenoids has also been described in children with bilateral VCP, further narrowing the airway in inspiration [17].

Despite enhanced glottic visualization, VL may offer false reassurance in these patients. Vocal fold immobility, midline fixation, and subglottic narrowing are not always evident on initial view, contributing to failed intubation attempts and misplaced confidence in the airway [4,9]. Repeated instrumentation risks worsening nerve injury, especially when the paralysis is partial or unrecognized [11].

Extubation presents a separate set of challenges. Reduced glottic closure, impaired laryngeal sensation, and poor reflexive protection increase the risk of aspiration, laryngospasm, and stridor [2,15]. In bilateral cases, respiratory decompensation may occur rapidly, particularly if pre-extubation assessment or prophylactic steroids are not used. Yet, no extubation protocols specific to VCP currently exist.

Surgical interventions such as cordotomy, arytenoidectomy, and suture lateralization may restore airway patency, but often at the expense of voice quality and with variable aspiration risk [14,17,18]. In select patients, chemodenervation with botulinum toxin has been proposed as a temporary method to suppress aberrant adductor activity and facilitate improved airflow [12].

Preoperative assessment and planning

Voice changes should be meticulously inquired about when taking a preoperative history. Hoarseness, breathy voice quality, or inspiratory stridor have been identified as common symptoms prior to presenting with critical upper airway obstruction secondary to VCP [19-21]. There is a consistent body of literature establishing an association between prior thyroid, anterior neck, or neck surgeries with prolonged intubation and recurrent laryngeal nerve injury leading to glottic dysfunction [19,20]. Although unilateral cord immobility may be well-tolerated, inspiratory airflow reserve may be suboptimal from associated compensatory glottic narrowing [19,20]. Not only may these patients be at increased risk for difficult intubation, but laryngoscopy can result in airway trauma and swelling [19]. The stability or progressive nature of the paralysis should be considered from the history and surgical record, particularly in determining the need for an awake intubation or timing of extubation [20,21].

In addition to history, there are important physical exam findings that may suggest cord paralysis. Inspiratory wheeze at rest can suggest bilateral cord immobility with limited ability to abduct, which is at risk for severe airway obstruction and respiratory depression when placed under sedation [19,20]. Voice evaluation may also help narrow the differential diagnosis. For example, breathy voice quality is consistent with poor glottic closure, while strained phonation may be more related to compensatory abduction [20,21]. Nevertheless, it is important to obtain a confirmatory evaluation with flexible endoscopic laryngoscopy when in doubt or when there are other high-risk features, such as a history of prior neck surgery or persistence of the above symptoms [20,21]. When formal laryngoscopy is not readily available, recent evidence suggests transcutaneous laryngeal ultrasound as an alternative non-invasive screening modality with high diagnostic performance when performed by experienced operators [22].

Once vocal cord immobility is confirmed, airway planning and strategy should be modified based on airway anatomy and function. Awake fiberoptic intubation is the mainstay in the management of bilateral VCP and cords that are immobile and fixed near the midline, as it allows for maintenance of airway reflexes while providing real-time visualization [21]. VL may improve glottic view in many cases, but given the distorted glottic geometry, it cannot reliably improve visualization in patients with VCP, and may result in malposition of the endotracheal tube (ETT) in the scenario of fixed cords [21]. In the setting of a vocal fold implant, aggressive or premature manipulation should be avoided due to the risk of displacement and mucosal trauma. Small internal diameter tubes, soft-tip stylets, and alternative devices can be used to limit these complications and may be associated with higher first pass intubation success and fewer perioperative airway complications in these patients [21].

In patients at highest risk for difficult airway and airway failure, such as those with bilateral VCP, history of previous vocal cord reconstruction, or chronic glottic insufficiency, it has been found that having a multidisciplinary plan with defined roles, having time for troubleshooting and run throughs, and backup plan such as surgical airway access, results in a significant benefit in safety and decreased morbidity [20,21]. This requires more formalized communication and coordination between anesthesia, otolaryngology, and surgery, but has been associated with successful airway management in challenging scenarios. Institutions with dedicated difficult airway registries and the routine performance of difficult airway scenarios on simulation have reported decreased rates of unanticipated intubation failure and higher provider confidence [21]. Perioperative anticipatory medical management with steroid prophylaxis for edema and planning for early postextubation monitoring may further increase safety in this population [21].

Intubation technique and first-pass success

When it comes to intubation, the VL is an exceptional instrument for improving visualization of the vocal cords and first-pass success rate. The process of using VL generally consists of inserting the device into the mouth, visualizing the airway on a screen, and then guiding the ETT into the trachea. Despite VL being a remarkable technique, there is a potential blind spot at the pharynx where the camera’s visual field loses sight of the tip of the ETT until the laryngeal inlet is met [23]. Intubation heavily relies on the correct positioning of the ETT to minimize complications in airway management, and a limited field of view, even for a moment of the ETT, significantly increases the chances for barotrauma, hypoxia, and even perforations. Additionally, VL has the capacity to visualize vocal cords well; however, upon insertion and placement, the acute angle formed by the blade makes it difficult to advance the ETT [24]. Good visualization does not mean successful intubation, and complications such as VCP have the potential to make the mechanical and anatomic challenges of proper tube placement increase the risk of adverse effects. Similarly, a different study revealed a higher utilization of ETT introducers with VL, and that improving glottic view may only matter to inexperienced operators [25]. Thus, VL may be one of the most common modern forms of intubation, yet it still encounters challenges associated with ETT placement. As VL continues to be integrated into modern medicine, it is critical for healthcare professionals to take into consideration the limitations of tube advancement caused by blade angulation, loss of the ETT tip visualization, and the potential cause for trauma.

Airway management with pre-existing conditions has to be constantly taken into consideration in anesthesia care. Medical concerns for securing the airway, such as unilateral VCP, increase the risk of anatomical and structural difficulties associated with intubation. In a case study by Song and Kim, a patient with left VCP underwent VL, but a proper laryngeal view was unobtainable [26]. Without direct observation or an adequate view of the airway, laryngeal deformities may displace the paralyzed vocal cord or misalign with the glottic opening, which can cause soft tissue trauma and an unsuccessful ETT insertion. Comparatively, these complications are supported in a case series from Santha et al., where three patients with unilateral VCP presented with adducted cords and a narrowed glottic aperture causing airway compromise [27]. In cases where VCP affects phonation and impairs airway integrity, alternative approaches such as awake fiberoptic intubation may provide greater control and safety. With pre-existing unilateral VCP, it is also vital to maintain mobile cord function to prevent other issues, such as airway edema, when focusing on anesthetic care [2]. Airway edema is another clinically significant worry when performing intubations, as it can continue to decrease the area in an already partially obstructed glottic space. As a result, it is essential to seek alternative methods that minimize further airway compromise and ensure patient safety.

Bilateral VCP is an additional concern in other areas of airway management despite a shared pathophysiology with unilateral VCP. In bilateral VCP, both vocal cords are affected, with the potential to cause limited airflow, trauma, and a high risk of airway obstruction, which greatly affects first-pass intubation. However, sometimes bilateral VCP is not truly noticeable until intubation. For example, in a case report by Doo et al., the patient underwent a laryngoscopy revealing flaccid bilateral paramedian vocal cords with an extremely narrow glottic gap that remained persistent even after the administration of additional rocuronium [28]. An unrecognizable bilateral VCP proposes unexpected risks with the potential of decreasing first-pass ETT insertion rates while also emphasizing the importance of preoperative airway assessment. Supportively, bilateral cord paralysis is known to extensively narrow the glottic opening, ultimately increasing the chance of traumatic insertion of the ETT and often requiring emergent airway interventions such as tracheostomies and laryngeal reinnervation [29]. Given these risks, such cases require modified strategies to maintain airway stability. In general, fixed vocal cords caused by bilateral VCP also result in increased susceptibility to pressure-related injuries during intubation [27]. Consequently, fixed vocal cords have proven to be problematic and thus warrant consideration during preoperative respiratory evaluation. Although uncommon, bilateral VCP poses challenges to securing the airway while also increasing the risk of perioperative complications, which ultimately encourages precautionary steps to ensure patient safety.

Securing ventilatory access may be compromised by narrowed or distorted anatomical and structural anomalies, which consequently also may affect ETT placement success rates. In an observational study by Takahashi et al., they revealed that patients with an airway obstruction had a lower first-pass success rate at 63% on average in comparison to 74% in non-obstructed patients [30]. Despite being a very common and essential medical procedure, there is a margin for error, especially in cases where the lumen is constricted. VCP, causing narrowing of the ventilatory space and immobilizing the glottis, ultimately alters the airway and is potentially a contributor to lowered success rates. In support of the previous findings, although specific research on VCP initial success intubation rate is limited, broader evidence of airway predictors reveals that chronic or acute disruptions of upper respiratory tract anatomy are strongly associated with reduced first-pass intubation rates [31]. VCP’s effect on laryngeal anatomy causes abnormalities that lower the chances of ETT insertion success, which is concerning because maintaining a secure airway is necessary for decreasing adverse outcomes. Additionally, VCP further complicates establishing a secure ventilatory route after an intubation failure because physiologic deterioration is exacerbated when intubation requires more than one attempt [32]. To minimize procedural complications that involve anesthesia, successful intubation with limited attempts is vital because not only do unnecessary intubation attempts increase the risk of unfavorable outcomes, but they may also induce more anatomical problems, such as perforations or trauma. First-pass intubation success is often overlooked because of its low failure rate; however, with complications such as VCP, securing the airway can be more challenging than it appears.

With modern medical advancement, there is ongoing research with methods that help deal with resolving the challenges of narrowed glottic lumens and air passage obstructions, which are common problems of VCP. Modifications in ETT sizing, bougie assistance, and gentle maneuvers and techniques may prove to have a beneficial effect. In a study by Maresch, they emphasized atraumatic intubation, smaller ETT sizes, and considered peri-op corticosteroids for facilitating airway access [2]. Prevention of complications during intubation is crucial because, despite being sensitive and delicate, laryngeal structures have a primary role in the respiratory system. Concerning ETTs explicitly, a study demonstrated that a smaller tube, such as 5.0 mm ETTs in contrast to larger tubes, was found to be safer and effective in limited laryngeal spaces [33]. The decrease in ETT diameter allows for adjustment room in the passage, ultimately decreasing the potential risk of perforation and trauma, making it effective for situations with restricted airway passages. A smaller ETT size selection is also useful in the prevention of intubation-related acute laryngeal injury [34]. Proper ETT sizing can be the difference between preserving respiratory integrity and losing safe airway access. In instances of restricted lumens, a smaller diameter ETT offers more maneuverability in challenging ventilatory passages, decreasing the risk of unsuccessful intubation attempts. Furthermore, bougies have also been shown to be effective in improving first-pass ETT insertion success in narrowed airway scenarios with poor glottic views [35]. Rather than attempting direct intubation in restricted airways, bougies, also known as ETT introducers, create a clear-cut pathway for the ETT, providing a safer and controlled approach. Current modern clinical practice for the utilization of bougie assistance and ETT selection is widely variable at this time, and with a lack of established protocols, there is a need for further exploration and research.

Intraoperative considerations and monitoring

Patients who suffer from VCP face significant intraoperative airway challenges, which require delicate planning and thorough monitoring to ensure the patient has safe ventilation and extubation. One risk associated with this clinical population is the danger of airway edema [36]. Specifically, post-intubation laryngeal edema, which is a pathological process that begins after intubation, and becomes clinically distinct due to causing respiratory difficulty after removal of the ETT [36]. Typically, post-intubation laryngeal edema is asymptomatic or mildly symptomatic. However, two out of three patients who present with post-extubation stridor have decreased vocal cord mobility, which results in nearly 50% of those patients being re-intubated shortly after [36]. The presence of VCP exacerbates this risk due to reduced mobility, making airway obstruction more likely.

Impaired vocal cord function additionally brings forth the need to consider risks such as pooling of secretions, stridor, and a diminished cough reflex. Stridor is a sign of inflammation in the larynx [37]. The presence of stridor after extubation may point to problems with vocal cord movement, vocal fold paralysis, or damage to mucosal tissue, such as ulcers or granulation [37]. This translates to emphasizing the importance of airway protection consideration during intubation and extubation, as there is an inverse relationship between cough strength and pneumonia risk, which is well established [38]. Meaning the stronger a patient can cough, the lower their risk of developing pneumonia post-operatively. Contrarily, the weaker a patient’s cough, the higher the risk of a patient developing pneumonia. VCP becomes a risk factor for aspiration pneumonia, which in turn increases postoperative morbidity and mortality as well [39]. Given the nature of VCP, patients are at risk of aspiration and pneumonia due to being unable to clear secretions by coughing. Adding to this complexity, certain risk factors such as geriatric patients' age and pre-existing comorbidities such as chronic pulmonary and cardiovascular diseases have been identified to increase the occurrence of VCP [39]. It is important to pay attention to these specific risk factors, as they may cause complications.

The importance of continuous monitoring of ventilation pressure and airway resistance is extremely important in VCP patients. Vigilant intraoperative observing by providers is critical due to the seemingly minor changes potentially being a signal to developing airway compromise [40]. This was demonstrated in a glossectomy case, where ventilation was managed using a volume control mode that had defined ventilation parameters and maintenance of peak airway pressures [40]. This level of precision is necessary in order to identify, address, and prevent any possible respiratory distress or complications. If ventilation parameters indicate airway compromise, ventilation pressures rise, or resistance in the airway increases, there are protocols present to address possible issues [40]. If these complications arise, examining for possible causes is essential; possible causes can include edema, tube misplacement, or the development of bilateral VCP. Recognizing and immediately addressing possible causes by potentially reintubating the patient or performing a tracheostomy can be effective to prevent any catastrophic airway loss [40]. Ultimately, readiness to intervene and monitor in real-time can support proactive responses and keep patients with vocal paralysis safe during procedures.

Additionally, thorough cuff tube management is important to reduce airway-related risks. The usage of cuff tubes is believed to support the prevention of aspiration [40]. Usage of cuff tubes can support patients with impaired glottic closures, as they are vulnerable to aspiration-related complications as well as postoperative discomfort [40]. This principle can additionally be applied to patients with VCP, as they also have impaired mobility and are similarly at risk for aspiration. Additionally, cuff pressure monitoring is essential to maintain the safety of patients and minimize the risk of cuff overinflation [21]. Some expert guidelines emphasize the necessity of maintaining continuous monitoring of cuff pressure, as overinflation with a syringe can raise pressures and volume to dangerous levels, which can compromise airway safety and vocal fold structure [21]. However, it is important to be aware that additionally overinflation of the cuff may result in recurrent laryngeal nerve injury; therefore, it is not recommended to solely rely on subjective palpation to assess for overinflation [40]. Instead, the usage of a manometer is recommended to monitor and control cuff pressures to remain at safe levels with repeat assessments to ensure constant examination [40]. These tools are extremely valuable to patients with VCP; however, they require attentiveness and observation in order to be used safely.

For patients with unilateral VCP, intraoperative neuromonitoring of the recurrent laryngeal nerve can be utilized to preserve vocal cord function. The usage reduces the risk of nerve injury during surgical procedures related to the larynx and airway [41]. This form of monitoring can preserve remaining vocal cord function for patients who have partial paralysis. Regardless of neural integrity being preserved, improper reversal of neuromuscular blockers can compromise airway patency. Therefore, there is an essential emphasis on neuromuscular monitoring, as well as ensuring neuromuscular blockades are fully reversed, before extubating patients VCP [42]. In regard to neuromuscular monitoring, it is recommended that clinicians use the adductor pollicis muscle. With consideration to medications, if a steroidal neuromuscular blocking agent has been administered, sugammadex can also be utilized by physicians as a reversal agent instead of the commonly used neostigmine [42]. The degree of neuromuscular blockade is commonly assessed using the train-of-four (TOF) monitoring method, which involves accounting for muscle twitch responses that are generated by nerve stimulation sequences.

A patient's recovery is evaluated based on the count of twitches, as it acts as an independent guide of neuromuscular function. However, for patients with VCP, TOF monitoring at a peripheral site should be used to objectively confirm complete reversal of neuromuscular blockade before considering extubation, as a TOF ratio of ≥0.9 indicates adequate global neuromuscular recovery [42]. However, because VCP poses a direct risk to airway patency and protective reflexes, clinicians must supplement TOF findings with direct airway assessment, such as laryngoscopy or a cuff leak test, and ensure vigilant postoperative monitoring. Preparation for emergency airway management and multidisciplinary collaboration are also essential in this high-risk group to ensure safe extubation and optimal recovery [40,43]. In usual practice, the degree of neuromuscular blockade is assessed by utilizing the TOF monitoring method.

This method involves counting muscle responses that are generated by nerve stimulation sequences. This count of muscle twitches provides an independent and otherwise objective guide for physicians to understand where a patient is in their stage of recovery and neuromuscular function [42]. However, due to VCP, patients are at an increased risk for loss of airway patency; therefore, TOF findings alone are not going to be satisfactory. In this population, clinicians must incorporate TOF monitoring with assessments such as cuff leak testing, laryngoscopy, and maintaining real-time postoperative monitoring [40,43]. In addition, being prepared with protocols for emergency airway management is essential to provide risk management and the best possible outcomes for extubation and recovery of patients with VCP [40,43]. Ultimately, combining active attention and preparation of protocols will provide the most optimal outcomes for patients (Table 1).

**Table 1:** Airway Management Techniques and Device Options in Patients With Vocal Cord Paralysis (VCP) ETT: endotracheal tube

Technique/Device	Benefits	Potential Risks	Clinical Indications
Awake fiberoptic intubation	Maintains reflexes, real-time navigation around fixed cords [21]	Requires topicalization and an experienced operator [20,21]	Bilateral VCP, fixed midline cords, prior laryngeal surgery/implant [21]
Video laryngoscopy	Improves view of glottis [23-25]	Tip blind spot, acute blade angle, false reassurance [4,9,23-25]	Unilateral VCP with acceptable aperture; bougie ready [2]
Bougie/introducer	Creates a path through a narrow gap to guide ETT [35]	Excess force can injure the mucosa if pushed [35]	Poor glottic view or tight posterior gap [35]
Smaller ETT (e.g., 5.0-6.0 adult)	More maneuver room, less trauma in the tight glottis [33,34]	Higher resistance; adapters may be needed [33,34]	Tight glottis, implants, high edema risk [33,34]
Steroid prophylaxis	Reduces post-extubation edema/stridor risk [44,45]	Timing and dosing vary by context [44,45]	High-risk extubations; borderline cuff-leak [44,45]
Cuff-leak test	High specificity for edema risk [46]	Moderate sensitivity; not stand‑alone [46]	Use with clinical exam and readiness plan [46]

Extubation and postoperative airway management

Extubation in patients with VCP, particularly bilateral VCP, presents with heightened risk due to the combination of a narrowed glottic aperture, impaired laryngeal sensation, and weakened cough reflex. These factors limit airway protection and clearance, especially under the stress of postoperative inflammation. When both vocal cords remain fixed near the midline, even minor edema may suffice to obstruct airflow completely. Thus, pre-extubation laryngeal evaluation using flexible endoscopy, VL, or direct visualization is essential to differentiate between unilateral and bilateral VCP. Such risk stratification directly supports the goals (as stated in the Abstract), namely safer airway planning and reduced perioperative complications. Tailoring the extubation strategy based on glottic findings can prevent emergent airway events and align with multidisciplinary planning protocols [44-46].

In patients identified as high risk, such as those with bilateral VCP, the use of prophylactic corticosteroids demonstrates measurable benefit in preventing post-extubation stridor and reducing morbidity. In the landmark trial by Lee et al., multiple-dose dexamethasone administration beginning 12-24 hours before extubation lowered stridor rates from 27.5% in placebo to 10%, though reintubation rates did not diverge significantly [44]. Complementing this, a network meta-analysis showed that both dexamethasone and methylprednisolone lead to significantly lower odds of stridor (odds ratio (OR) ≈ 0.39 and 0.22) and reintubation (OR ≈ 0.34 and 0.42) compared to hydrocortisone or placebo [45]. Taken together, these findings support targeted pre-extubation anti-inflammatory therapy to preserve glottic function and reduce the need for emergency airway intervention in vulnerable surgical populations.

The cuff-leak test (CLT) is an established method for predicting airway edema before extubation, though its utility hinges on understanding its diagnostic limitations. In a meta-analysis of 28 adult studies across more than 4,400 extubations, the CLT displayed excellent specificity (~87-88%) but only moderate sensitivity (~62%) for detecting post-extubation airway obstruction or need for reintubation [46]. In clinical practice, a low cuff-leak volume (e.g., <110 mL or <10% of tidal volume) strongly indicates risk and may justify steroid administration or delaying extubation. However, a normal CLT does not preclude adverse outcomes, particularly in anatomically compromised patients. Therefore, the CLT should be incorporated as one element of comprehensive risk assessment rather than a sole decision-making criterion.

Safe extubation demands rigorous patient readiness: the individual must be fully awake, exhibiting adequate neuromuscular recovery (TOF ratio ≥ 0.9), sustaining spontaneous ventilation, and capable of protecting their airway. Positioning with the head elevated helps optimize pulmonary mechanics and reduce airway resistance during emergence. Clinicians should ensure immediate access to airway adjuncts, including suction catheters, soft-tip stylets, and alternative tube sizes, as well as reintubation supplies. The onset of post-extubation stridor, especially if combined with desaturation or accessory muscle use, should prompt immediate reintubation rather than observation to prevent rapid respiratory failure. These measures align with ICU-level monitoring and ENT backup in critical VCP cases [44-46].

Beyond traditional evaluation tools, innovative methods like the “pressure above the cuff” measurement may provide a more refined airway patency assessment. In a pilot study by Tokunaga et al., this technique strongly correlated with cuff-leak volumes (r = -0.76, p < 0.001) and avoided ventilator asynchrony that can occur with cuff deflation [47]. Though clinical outcomes such as stridor or reintubation were not yet assessed, the method shows promise for minimizing testing-related airway disturbance in high-risk patients. Coupled with emerging modalities, such as laryngeal ultrasound to assess air‑column width, these approaches suggest an evolving toolkit for more precise extubation planning in patients with vocal cord dysfunction. Table 2 summarizes unilateral versus bilateral VCP features and perioperative implications.

**Table 2:** Key Airway Implications and Management Strategies in Unilateral and Bilateral Vocal Cord Paralysis (VCP) VL: video laryngoscopy; ETT: endotracheal tube; TOF: train-of-four

Domain	Unilateral VCP	Bilateral VCP
Anatomy and Physiology		
Cord position	One cord abducted or paramedian; compensatory closure may narrow aperture [1,13,19-21]	Both cords median/paramedian; fixed very small glottic gap [7,14]
Airflow impact	Partial narrowing; turbulence increases with sedation; aspiration risk due to incomplete closure [2,15,16]	High resistance; minor edema can fully obstruct; baseline stridor risk [7,14]
Recognition and Pre-op Assessment		
Clinical cues	Hoarseness; breathy voice sometimes normal at rest; worsens with manipulation [19-21]	Inspiratory wheeze/stridor at rest; low exercise tolerance [19-21]
Confirmatory tests	Flexible laryngoscopy or laryngeal ultrasound when available [20-22]	Same; consider preop ENT consult [20-22]
Intubation Strategy		
Preferred approach	VL or fiberoptic acceptable; use smaller ETT with gentle technique; bougie available; avoid implant trauma if present [2,4,9,21,23-25,33-35]	Awake fiberoptic first-line small ETT; soft-tip stylet; have surgical airway plan; ENT at bedside [2,21,33,34,40]
Extubation Strategy		
Readiness and risk control	Usually feasible with mitigation cuff-leak + clinical exam; steroids if borderline risk [44-46]	Often delayed or staged in ICU; prophylactic steroids when appropriate; low threshold to reintubate or tracheostomy [2,40,43-45]
Cross-Cutting Safety		
Monitoring and precautions	Neuromuscular block fully reversed (TOF ≥ 0.9); cuff pressure monitored with manometer [21,40,42]	Same as unilateral; plan for ICU monitoring in high risk [43]

Gaps in the literature and future research directions

VL is increasingly used in critical care settings, although its role specifically in VCP remains unexplored, indicating a gap in available research. Araújo et al. conducted 14 randomized controlled trials (RCTs) with a total of 3,981 patients, demonstrating that VL improves first attempt intubation success rates while lowering both esophageal intubation and aspiration risks when compared to direct laryngoscopy (DL) [48]. However, this analysis concludes broad populations of critically ill patients while neglecting the unique subgroup of VCP patients characterized by limited glottic mobility and often unpredictable airway behavior. The implication drawn from this analysis is that while VL offers high-caliber visualization and maneuverability, anatomical distortions or flaccid folds with VCP patients represent challenges that the general RCTs fail to account for. Furthermore, the study conducted by Hannah et al. in a pre-hospital environment used VL versus DL to analyze real-world outcomes without distinguishing patients with underlying VCP [49]. This absence accentuates the issue that not only are VCP patients poorly represented, but they are often not stratified in airway research, diluting the applicability of findings. The study by Yuan et al. further confirms the advantages of VL in critical illness among adults through systematic review results, as it emphasizes its potential to improve outcomes [50]. Still, they too fall short in addressing sufficient attention to patient-specific factors. This includes factors that influence glottic visualization and tube passage, along with airway trauma risks because of vocal fold immobility. These studies indicate favorable VL outcomes compared to DL, yet fail to definitively establish best practices for managing VCP patients. This leaves clinicians to draw from diverse untargeted data rather than evidence tailored to the unique traits of this subgroup. This emphasizes a critical research need to directly evaluate laryngoscopy approaches in the context of VCP using targeted and prospective study designs that recognize the anatomical and physiological challenges of this unique population.

The lack of standardized clinical protocols for intubation and extubation procedures in VCP patients further contributes to the clinical marginalization of VCP. Standardized clinical protocols are absent, which creates variable procedural management approaches that could increase laryngeal injuries and compromise airway stability and prolong complication detection. Lim et al. reviewed 43 cases to show that recurrent laryngeal nerve injury was a common complication following surgery because of long intubation times, improper ETT sizes, and inadequate patient positioning [51]. Decision-making in these cases shows strong dependence on operator expertise because there exists no standardized protocol. Jung further demonstrates that successful airway management depends on precise context-specific planning when patients have altered airway anatomy. The process of extubation in these patients needs more than standard criteria; it requires formal evaluation of vocal fold movement along with assessment of airway clearance and edema risk, yet the standard currently lacks any standardized framework for this evaluation level [52]. The study by Elmer et al. demonstrates that premature or unprepared extubation leads to increased rates of aspiration and hypotension and higher ICU mortality among critically ill patients [53]. The risk of permanent damage becomes more significant because VCP patients need to undergo repeated airway procedures. Airway management of VCP patients lacks evidence-based protocols, resulting in unpredictable and dangerous practices, according to the research findings. The development of standardized intubation and extubation pathways that incorporate multidisciplinary evaluation for VCP patients stands as a fundamental requirement for advancing safe perioperative care.

Following the concern regarding the absence of standardized protocols for intubation and extubation in VCP, the lack of clarity surrounding optimal perioperative steroid strategies and ETT sizing raises an equally pressing issue. Despite the common use of corticosteroids for preventing laryngeal edema and post-extubation complications (especially in patients with fragile or compromised airways), there are no established guidelines for steroid administration in VCP. According to Seo, dexamethasone is frequently given pre-extubation, but its effectiveness remains unclear because of the inconsistent results across patient groups and different extubation protocols. Extubation of patients with VCP becomes more dangerous because minor mucosal swelling reduces glottic aperture, while the uncertainty increases re-intubation or tracheostomy rates. The post-intubation outcomes of these patients depend directly on selecting appropriate ETT sizes, which is an often-neglected factor [54]. The study by Esianor et al. (2022) revealed that oversized ETTs used in critically ill patients resulted in higher vocal fold immobility rates because they caused iatrogenic trauma to patients without pre-existing laryngeal dysfunction. VCP patients would face increased glottic injury because of improper tube selection, yet there exists no established standard for choosing tube diameter based on airway pathology [55]. Additionally, modern preoperative airway assessments such as ultrasonography and CT imaging are advancing, yet Marchis et al. (2024) emphasized that these tools are not effectively used in intraoperative decision-making. A gap exists between diagnostic understanding and procedural planning, which could increase VCP-related complications [56]. The combination of non-standard steroid administration, unpredictable tube diameter choices, and insufficient airway assessment indicates a wider problem, which is the absence of proven VCP-focused perioperative management protocols. To address this gap, future research must involve purposeful prospective studies that focus specifically on VCP-related risks, rather than general critical care populations, and assess how various steroid dosing and airway equipment sizing affect patient outcomes.

The existing deficit of information regarding long-term airway results after multiple intubations represents another critical challenge. Current clinical focus prioritizes immediate airway management and procedural success, while the effects of repeated intubation procedures over time remain poorly defined. Jung et al. examined functional recovery in ischemic stroke patients managed via extubation attempts versus primary tracheostomy and found no significant differences in one-year outcomes between extubation failure and extubation success groups, although primary tracheostomy patients had a worse prognosis overall [57]. Hurtado Nazal et al. presented a series of cases in which patients developed VCP after intubation, and presented that even isolated intubation events can lead to VCP through nerve damage and scarring that worsen with repeated airway interventions [58]. Meanwhile, Küçük et al. observed that patients who have been intubated before often present with latent laryngeal pathologies. This only became apparent during evaluation for unrelated complaints, thus demonstrating health complications that standard perioperative evaluations were unable to detect. These findings point to a critical and large void in longitudinal research, which is the lack of focused research on post-discharge airway monitoring of patients. VCP patients need multiple intubations, creating uncertainty about long-term risks [59]. The discovery of this knowledge gap serves two essential purposes: it protects patient safety and enables the development of future guidelines that focus on long-term airway health instead of just short-term procedure success.

## Conclusions

VCP presents a high-risk but ultimately controllable airway challenge when predicted and appropriately treated. Optimal outcomes depend on early identification, comprehensive preoperative assessment, and adaptation of airway techniques to the patient's glottic anatomy and functional status. While VL offers enhanced visualization, its utility must be balanced with clinical judgment, as it may offer false reassurance in cases of immobile or narrowed vocal folds. Equally important is the extubation strategy, which requires pre-extubation laryngeal evaluation, steroid prophylaxis, and readiness for reintubation or surgical airway. The absence of VCP-specific extubation protocols and inclusion in national airway guidelines reflects a critical gap in current practice. Resultingly, further research is needed to inform the development of standardized airway algorithms for this vulnerable and endangered population.
